# Knowledge, Attitudes, and Preventive Practices Related to Hepatitis B Infection Among Adults in Saudi Arabia: A Nationwide Cross-Sectional Study

**DOI:** 10.3390/healthcare14111558

**Published:** 2026-06-02

**Authors:** Mohammad A. Jareebi, Ghazi I. Al Jowf, Saja A. Almraysi, Dhiyaa A. H. Otayf, Khalil I. Hakami, Wesam H. Aridhi, Abrar Fahad Alshahrani, Omar Oraibi, Mostafa Mohrag, Sameer Alqassimi, Saleh A. Almazam, Khalid S. Alsallumi, Zakaria I. Melaisi, Majed A. Ryani, Farjah H. Algahtani

**Affiliations:** 1 Department of Family and Community Medicine, College of Medicine, Jazan University, Jazan 45142, Saudi Arabia; majedryani@gmail.com; 2 Department of Public Health, College of Applied Medical Sciences, University Medical Clinics Complex, King Faisal University, Al Hofuf 37912, Saudi Arabia; 3 Faculty of Medicine, Jazan University, Jazan 45142, Saudi Arabia; sajaalasiri1@gmail.com (S.A.A.); dhiyaaot@gmail.com (D.A.H.O.); khalil.hakami21@gmail.com (K.I.H.); wesam.aridhi@gmail.com (W.H.A.); 4 Department of Clinical Nutrition, College of Nursing and Health Sciences, Jazan University, Jazan 45142, Saudi Arabia; afalshahrani@jazanu.edu.sa; 5 Department of Internal Medicine, College of Medicine, Jazan University, Jazan 45142, Saudi Arabia; ooraibi@jazanu.edu.sa (O.O.); mmohrag@jazanu.edu.sa (M.M.); smalqassimi@jazanu.edu.sa (S.A.); 6 Pharmacology and Toxicology Department, College of Pharmacy, Umm Al-Qura University, Makkah 21955, Saudi Arabia; samazam@uqu.edu.sa; 7 Pharmaceutical Practices Department, College of Pharmacy, Umm Al-Qura University, Makkah 21955, Saudi Arabia; kssallumi@uqu.edu.sa; 8 Jazan Health Cluster, Jazan 45142, Saudi Arabia; zak.abb@gmail.com; 9 Oncology Center, Chair of Epidemiology and Public Health Research, Faculty of Medicine, King Saud University/King Saud Medical City, Riyadh 12373, Saudi Arabia; falgahtani@ksu.edu.sa

**Keywords:** hepatitis B infection, knowledge, attitudes, and practices (KAP), preventive practices, vaccination, screening, health education, Saudi Arabia

## Abstract

**Background**: Hepatitis B virus (HBV) infection poses a persistent global public health challenge, with substantial associated morbidity, mortality, and healthcare utilization. Although Saudi Arabia has maintained a national HBV vaccination program for decades, population-level data on hepatitis B infection knowledge, attitudes, and practices (KAP) remain scarce and regionally limited. This study aimed to comprehensively assess KAP toward hepatitis B infection prevention among the general adult population across all regions of Saudi Arabia and to identify independent sociodemographic predictors of each domain to inform targeted healthcare interventions. **Methods**: This nationwide cross-sectional study used a convenience sampling approach and a validated, self-administered questionnaire disseminated via online social media platforms across all regions of Saudi Arabia between August 2024 and February 2025. KAP was assessed using an instrument adapted from Haq et al. (Cronbach’s α = 0.70). Good knowledge was defined as a score ≥11/20 (≥55%), positive attitude as ≥5/7 (≥71.4%), and good practice as ≥6/8 (≥75%). Multivariable linear regression was used to identify independent predictors, adjusting for sociodemographic covariates. **Results**: A total of 1278 participants were included (mean age 30.3 ± 12.4 years; 60.9% female). Overall, 54.2% demonstrated good knowledge, 68.5% demonstrated positive attitudes, and only 16.2% exhibited good preventive practices. Screening (14.6%) and vaccination uptake (26.5%) were markedly low. Educational program participation was the strongest modifiable predictor across all three domains: knowledge (β = +1.89, 95% CI: 1.20–2.58, *p* < 0.001), attitude (β = +0.47, 95% CI: 0.25–0.69, *p* < 0.001), and practice (β = +1.43, 95% CI: 1.09–1.77, *p* < 0.001). Healthcare sector employment was independently associated with higher KAP scores across all domains. Income demonstrated a positive dose–response relationship with knowledge and practice outcomes. Polygyny was associated with lower scores across all three domains. **Conclusions**: Despite moderate knowledge and generally favorable attitudes, preventive practices remain critically deficient, revealing a persistent knowledge–practice gap. Integrated, behavior-oriented interventions targeting modifiable determinants, particularly health education, income disparities, and stigma, are urgently needed to support progress toward national and global HBV elimination targets.

## 1. Introduction

Hepatitis B virus (HBV) infection remains one of the most significant preventable causes of chronic liver disease worldwide. The World Health Organization (WHO) estimated that 254 million people were living with chronic HBV infection in 2022, with approximately 1.2 million new infections and 1.1 million deaths occurring annually, primarily attributable to hepatocellular carcinoma (HCC) and cirrhosis [1]. In recognition of this burden, the WHO Global Health Sector Strategy on Viral Hepatitis established ambitious targets for 2030, including a 95% reduction in new HBV infections and a 65% reduction in HBV-related mortality compared with 2015 baselines [2]. Despite these commitments, current trajectories fall considerably short, and intensified action across prevention, diagnosis, and treatment cascades is urgently required [1,2].

In Saudi Arabia, the epidemiology of HBV has undergone a marked transformation since the introduction of the universal infant vaccination program in 1989–1990, followed by school-based catch-up campaigns, which collectively produced a substantial decline in infection rates, particularly among children and adolescents [3,4]. Nonetheless, HBV has not been eliminated as an ongoing public health concern. A national analysis spanning 2006–2021 documented persistent regional variation in incidence, with a declining overall trend nationally but disproportionately higher rates persistently observed in western and northern regions [5]. In addition, several systemic challenges persist, including incomplete surveillance coverage, variable levels of population awareness, stigma associated with the diagnosis, and inequitable access to specialist care [6].

Knowledge, attitudes, and practices (KAP) toward HBV are recognized as critical determinants of both individual-level prevention and population-level transmission dynamics. The KAP framework posits that health behavior change follows a sequential pathway in which adequate knowledge forms the cognitive foundation for the development of favorable attitudes, which in turn facilitate the adoption of protective practices [7,8,9]. Adequate public knowledge of HBV transmission routes, vaccination availability, and the asymptomatic nature of early infection is essential for promoting timely health seeking behavior. Such awareness may also contribute to reducing stigma toward affected individuals [10]. Prior evidence from Saudi Arabia demonstrates heterogeneous KAP findings across different regions. While some studies have reported moderate to favorable levels of knowledge and attitudes [11,12], others have identified substantial gaps, particularly among non-healthcare populations [13,14]. Moreover, preventive practices, including vaccination uptake, participation in screening programs, and engagement in health education initiatives, remain consistently suboptimal [15,16]. Existing Saudi studies have been predominantly restricted to single regions or specific occupational groups, have relied on non-validated instruments, and have generally not employed multivariable analytical frameworks capable of isolating the independent contributions of sociodemographic factors to KAP outcomes [8,17,18]. These preventive behavior gaps are particularly concerning given Saudi Arabia’s status as a high-income country with established HBV care infrastructure.

Saudi Arabia’s Vision 2030 health transformation agenda explicitly prioritize preventive medicine, population health screening, and digital health innovation as foundational pillars of its healthcare restructuring [19,20]. HBV prevention represents a directly relevant domain within this framework, as improvements in vaccination coverage, screening uptake, and public health literacy align closely with Vision 2030’s national preventive medicine initiatives and its emphasis on leveraging digital platforms to enhance health awareness and service accessibility at the population level [21,22]. Therefore, this study aimed to address these gaps by providing the first multivariable assessment of hepatitis B infection KAP across all regions of Saudi Arabia using a validated instrument, with the specific objective of identifying independent sociodemographic predictors of each domain to inform targeted, scalable public health interventions.

## 2. Materials and Methods

### 2.1. Study Design and Eligibility Criteria

This cross-sectional study employed a convenience sampling approach using a self-administered questionnaire to assess knowledge, attitudes, and preventive practices related to hepatitis B infection among adults in Saudi Arabia. Trained data collectors were assigned to one of the major regions of the country (Central, Western, Eastern, Northern, and Southern). Each collector disseminated the survey link through region-specific social media networks to maximize geographic coverage. This strategy ensured representation from all regions of Saudi Arabia.

Participants were recruited through social media platforms, a method that may introduce selection bias and limit the representativeness of the sample relative to the broader Saudi adult population. Eligible participants were adults aged ≥18 years residing in Saudi Arabia who provided informed consent after reviewing a detailed explanation of the study objectives, potential risks, and participant rights. Individuals who did not complete the questionnaire were excluded from the final analysis.

### 2.2. Sample Size

Sample size was calculated using a 95% confidence level (Z = 1.96), an expected proportion (p) of 0.50 to maximize the required sample, and a margin of error (e) of 2.83%, applying the standard formula for cross-sectional surveys:


$$n_{o}=\frac{Z^{2}\cdot p\cdot (1-p)}{e^{2}}$$

This yielded a minimum required sample of 1200 participants. To enhance the precision of estimates, the final target sample was increased to 1278 respondents.

### 2.3. Data Collection Instrument

The data collection instrument was developed following a comprehensive review of the existing literature, with consideration of the Saudi sociocultural context and relevant risk factors. The draft questionnaire was reviewed by subject-matter experts, and revisions were made accordingly. A pilot study was subsequently conducted with 30 participants, and the instrument was further refined based on their feedback to improve clarity and cultural appropriateness. The 30 participants involved in the pilot study were excluded from the final analysis to ensure the integrity of the dataset.

The final questionnaire comprised four main sections. The first section captured sociodemographic characteristics, including age, sex, and region of residence, marital status, educational level, monthly household income, work sector, and smoking status. The second section addressed habitual and health-related characteristics. The third section assessed hepatitis B infection-related factors, including personal diagnosis, family history, and prior participation in educational programs. The fourth section assessed KAP toward hepatitis B using an instrument adapted from Haq et al., comprising 20 knowledge items, 7 attitude items, and 8 practice items, with demonstrated acceptable reliability and validity (Cronbach’s α = 0.70) [9,23]. Good knowledge was defined as a score ≥11 out of 20 (≥55%), positive attitude as a score ≥5 out of 7 (≥71.4%), and good practice as a score ≥6 out of 8 (≥75%), consistent with the scoring thresholds applied in the original instrument. These thresholds reflect the minimum proportions considered indicative of adequate performance in each domain and ensure comparability with prior studies utilizing the same instrument. The original instrument was translated into Arabic by a bilingual professional and subsequently back-translated into English by an independent translator to verify conceptual equivalence. Content validity was assessed through expert review, and construct validity was evaluated during the pilot phase.

### 2.4. Data Collection Process

Data were collected between October 2024 and February 2025 via an online self-administered survey. The questionnaire was disseminated by eight trained data collectors, each assigned to a specific region of Saudi Arabia to maximize geographic coverage. Data collectors received standardized briefing sessions covering study objectives, participant eligibility criteria, ethical conduct, and informed consent procedures. This multi-channel dissemination strategy aimed to recruit participants from all regions of Saudi Arabia. The survey included an introductory section describing the study objectives and participants’ rights. Responses were reviewed periodically for consistency, and incomplete questionnaires were excluded from the analysis. To minimize the risk of duplicate responses, each submission was linked to a unique device identifier, and responses sharing identical IP addresses or completing the survey in implausibly short durations were reviewed and excluded during the data cleaning process.

### 2.5. Statistical Analysis

Data were entered into Microsoft Excel for cleaning and verification prior to analysis. Statistical analyses were conducted using RStudio (version 4.2.3; R Foundation for Statistical Computing, Vienna, Austria). Means, standard deviations, and frequencies with proportions were used to summarize participant characteristics and KAP item-level responses. Three separate multivariable linear regression models were constructed to identify independent predictors of knowledge, attitude, and practice scores, respectively. Multivariable linear regression was selected to preserve the continuous, graded nature of KAP scores, to facilitate direct interpretation of predictor effect sizes across domains, and is consistent with analytical approaches adopted in comparable KAP studies in the literature [24]. All statistical tests were two-tailed, with the significance threshold set at α = 0.05, and 95% confidence intervals were reported throughout.

### 2.6. Ethical Approval

The study was approved by the Standing Committee for Scientific Research at Jazan University (REC-46/02/1167; 1 September 2024). All procedures followed institutional and national ethical standards and the principles of the Declaration of Helsinki and followed the Strengthening the Reporting of Observational Studies in Epidemiology (STROBE) guidelines to ensure methodological rigor and transparent reporting of this cross-sectional investigation.

### 2.7. Use of Generative Artificial Intelligence

Generative artificial intelligence tools were used exclusively for language editing and grammatical refinement. All study design, data collection, analysis, interpretation, and scientific conclusions were conducted independently by the authors.

## 3. Results

### 3.1. Sociodemographic and Health-Related Characteristics

**Figure 1:**
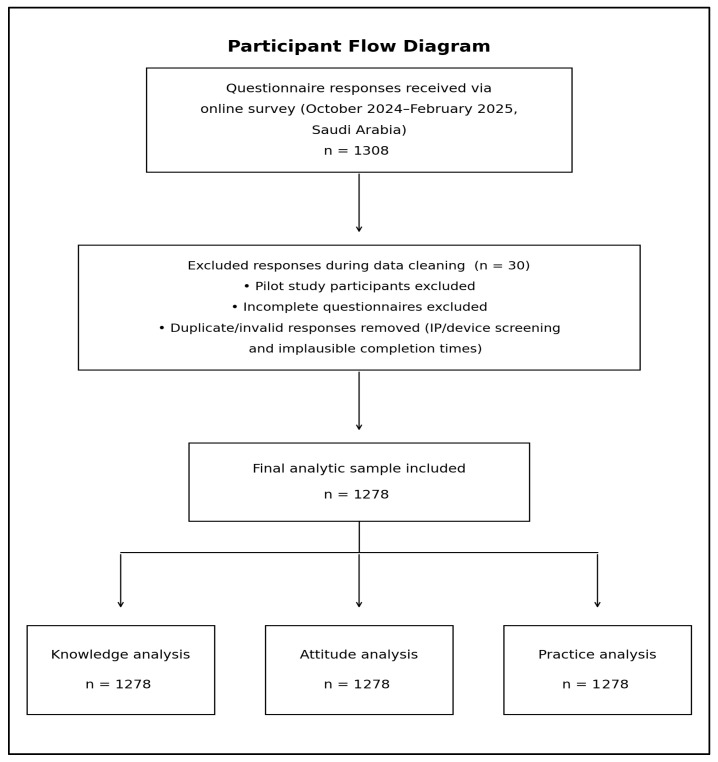
Participant flow diagram.

A total of 1278 participants were included (Figure 1). The mean age was 30.3 ± 12.4 years, and most participants were female (n = 778, 60.9%). The majority were Saudi nationals (n = 1196, 93.6%) residing in urban areas (n = 1132, 88.6%). The Western region contributed the largest proportion of respondents (n = 812, 63.5%), followed by the Southern (n = 179, 14.0%), Eastern (n = 136, 10.6%), Central (n = 115, 9.0%), and Northern regions (n = 36, 2.8%). More than half of the participants were single (n = 701, 54.9%), and the majority held a bachelor’s or diploma degree (n = 893, 69.9%). The largest income groups reported monthly earnings of ≥15,000 SAR (n = 365, 28.6%) and 10,000–14,999 SAR (n = 337, 26.4%). Regarding the work sector, participants were classified into four groups: students (n = 481, 37.6%), other employed (n = 474, 37.1%; comprising educational, military, administrative, industrial, handicrafts/freelance, and agricultural workers), healthcare sector (n = 167, 13.1%), and unemployed (n = 156, 12.2%). Most participants were never smokers (n = 1070, 83.7%). Complete characteristics are presented in Table 1.

### 3.2. Hepatitis B Infection Diagnosis, Family History, and Educational Program Participation

Table 2 presents hepatitis B infection-related characteristics. Only 30 participants (2.3%) reported a prior diagnosis of hepatitis B infection, and 108 (8.5%) reported a positive family history. Prior participation in a structured hepatitis B infection educational program was reported by 105 participants (8.2%). 

### 3.3. Knowledge of Hepatitis B Infection Among Participants

**Figure 2:**
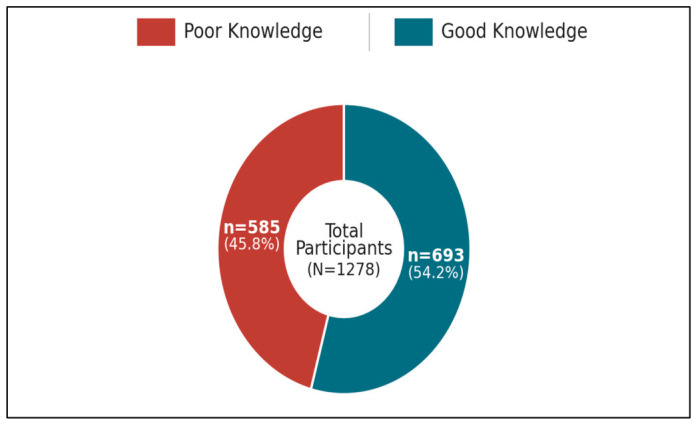
Distribution of knowledge toward Hepatitis B infection among participants (n = 1278).

Table 3 presents item-level knowledge responses. The knowledge questionnaire comprised 20 binary items (maximum score = 20); participants scoring ≥ 11 (≥55%) were classified as having good knowledge. General awareness was moderate: 76.6% had heard of hepatitis and 65.2% specifically of hepatitis B infection. Disease characterization was variable as 65.2% correctly identified that hepatitis B infection affects liver function, yet only 48.5% identified it as a viral disease and 37.8% knew it can cause liver cancer. Knowledge of symptoms was similarly inconsistent: while 79.9% recognized early cold/flu-like symptoms, only 48.0% identified jaundice and only 35.5% acknowledged that some patients may remain entirely asymptomatic. Awareness of transmission routes was suboptimal across all routes. The most frequently recognized were contaminated blood or blood products (51.8%) and unsterilized needles or surgical instruments (49.5%). Notably, mother-to-child transmission was recognized by only 32.9% of participants and transmission via unsafe sexual contact by only 38.4% two epidemiologically important routes. A total of 70.9% correctly identified that hepatitis B infection is not transmitted via contaminated food or water. Regarding prevention and treatment, 85.3% correctly indicated that the disease cannot be self-cured, 59.4% recognized it as treatable, and 55.1% were aware that vaccination is available. The mean knowledge score was 11.1 ± 3.7 (median: 11; IQR: 8–14). Overall, 693 participants (54.2%) demonstrated good knowledge and 585 (45.8%) demonstrated poor knowledge (Figure 2).

### 3.4. Attitudes Toward Hepatitis B Infection Among Participants

**Figure 3:**
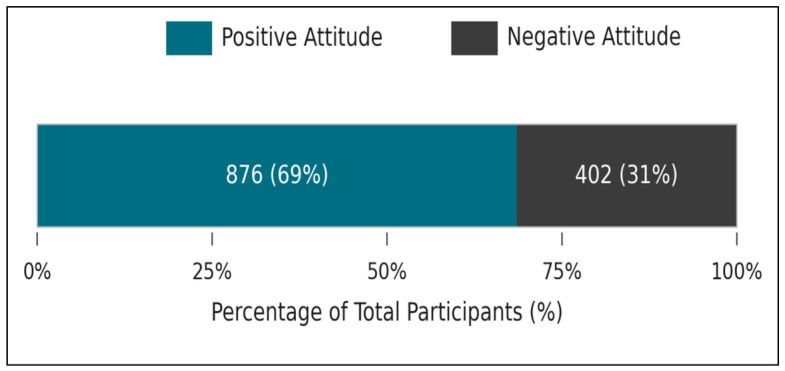
Distribution of attitudes toward Hepatitis B infection among participants (n = 1278).

Table 4 presents attitude item responses. The attitude scale comprised seven binary items (maximum score = 7); participants scoring ≥ 5 (≥71.4%) were classified as having a positive attitude. Despite generally favorable healthcare-seeking intentions, 90.8% would seek care at a health facility if symptomatic, and 76.7% who would do so promptly perceived susceptibility as markedly low, with only 186 participants (14.6%) believing they were at risk of contracting hepatitis B infection. Of those who would seek care, 65.7% specifically indicated they would consult a physician; accordingly, the disclosure item (a3) was coded as positive for any responsible disclosure, yielding 1185 positive responses (92.7%). Regarding perceived cost, 784 participants (61.3%) considered services free or reasonably priced, while 494 (38.7%) were unaware of associated costs. Fear of transmitting the infection to family members was endorsed by all participants (100.0%); as this item showed no variance, it was retained for descriptive purposes but excluded from regression analyses. The mean attitude score was 4.9 ± 1.1 (median: 5; IQR: 4–6), with 876 participants (68.5%) demonstrating a positive attitude and 402 (31.5%) a negative attitude (Figure 3).

### 3.5. Preventive Practices Related to Hepatitis B Infection

**Figure 4:**
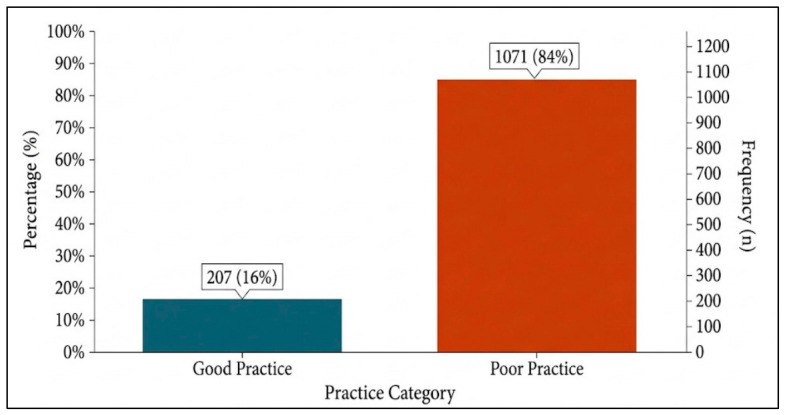
Practices related to Hepatitis B infection among participants (n = 1278).

Table 5 summarizes preventive practice responses across eight binary items (maximum score = 8); participants scoring ≥ 6 (≥75%) were classified as having good practice. Uptake of systematic preventive services was markedly low: only 186 participants (14.6%) reported prior hepatitis B infection screening and 338 (26.5%) reported vaccination. Situational preventive behaviors were comparatively more prevalent: 862 (67.4%) would request safe barbering or piercing practices, 840 (65.7%) would request a new syringe, and 733 (57.4%) would request blood product screening before transfusion. Most participants (n = 1000, 78.2%) would seek further investigation or treatment if diagnosed, and 733 (57.4%) reported they would not avoid contact with infected individuals. Health education engagement was limited, with only 166 participants (13.0%) reporting prior participation; this item assessed any health education engagement and is distinct from the structured HBV program participation variable in Table 2 (n = 105, 8.2%). The mean practice score was 3.8 ± 1.8 (median: 4; IQR: 2–5). Only 207 participants (16.2%) demonstrated good preventive practices, while 1071 (83.8%) were classified as having poor practices (Figure 4).

### 3.6. Multivariable Predictors of Hepatitis B Infection Knowledge, Attitudes, and Practices

Three multivariable linear regression models were constructed to identify independent predictors of KAP scores. Higher knowledge scores were independently associated with older age, married status, mid-to-high household income, healthcare sector employment, student status, educational program participation, and a positive family history of hepatitis B infection. Male sex, polygyny, and a personal hepatitis B infection diagnosis were each associated with significantly lower knowledge scores. Formal educational attainment was not independently associated with knowledge after adjustment. Higher attitude scores were independently associated with older age, married status, mid-range household income, healthcare sector employment, and educational program participation. Polygyny was the only variable independently associated with lower attitude scores. Higher practice scores were independently associated with older age, mid-to-high household income, healthcare sector employment, educational program participation, and a positive family history of hepatitis B infection. Male sex and polygyny were independently associated with lower practice scores. Student status, despite predicting higher knowledge, was not associated with practice scores. Complete estimates are presented in Table 6.

## 4. Discussion

This nationwide cross-sectional study investigated KAP toward hepatitis B infection and its independent sociodemographic predictors among adults in Saudi Arabia. The principal finding was a marked and clinically important knowledge–practice gap: although 54.2% of participants demonstrated good knowledge and 68.5% demonstrated positive attitudes, only 16.2% exhibited good preventive practices, with vaccination uptake (26.5%) and screening participation (14.6%) remaining critically low. These findings have direct implications for Saudi Arabia’s progress toward the WHO 2030 hepatitis B elimination targets. Vaccination uptake of 26.5% and screening participation of 14.6% are markedly insufficient by any benchmark, including the WHO target of ≥90% vaccination coverage.

### 4.1. Public Knowledge of Hepatitis B Infection

The moderate level of knowledge observed (54.2%) indicates that public understanding of HBV in Saudi Arabia remains suboptimal, despite a national vaccination program that has operated for over three decades [3]. This figure is broadly consistent with prior regional studies: Almalki et al. reported 61.2% good knowledge in the Makkah region [7], and Alotaibi et al. reported 55.1% in a separate Saudi sample [8], while a systematic review of Gulf Cooperation Council countries documented knowledge levels ranging from 40% to 60% [25]. Taken together, these data suggest that knowledge deficits have persisted without meaningful improvement over time.

Several specific knowledge gaps identified in the present study are of public health concern. Mother-to-child transmission was recognized by only 32.9% of participants, and transmission via unsafe sexual contact by only 38.4%—two routes that are central to sustained HBV transmission in adulthood. These gaps likely reflect inadequate targeted messaging around perinatal and sexual transmission beyond the childhood vaccination context. Furthermore, only 35.5% of participants acknowledged that HBV infection may remain asymptomatic, a critical misconception that may contribute to delayed testing and false reassurance among high-risk individuals. These findings align with a Jordanian study reporting similarly suboptimal recognition of transmission routes among students [26] and with the broader Gulf region evidence base [9].

### 4.2. Attitudes and Perceived Risk of Hepatitis B Infection

The majority of participants (68.5%) demonstrated positive attitudes, reflected particularly in strong healthcare-seeking intentions: 90.8% reported they would seek care at a health facility if symptomatic, and 65.7% would specifically consult a physician. These findings suggest a generally favorable disposition toward professional medical care among the Saudi population, which is encouraging for health system engagement. However, these positive intentions were sharply juxtaposed with markedly low perceived susceptibility: only 14.6% believed they were personally at risk of contracting HBV. This constitutes a major attitudinal barrier to preventive behavior. According to the Health Belief Model, perceived susceptibility is a primary driver of health-protective actions; in its absence, favorable knowledge and intentions are unlikely to translate into sustained behavioral change [6,27]. Within the established healthcare framework, the persistence of these behavioral gaps likely reflects the interplay of multiple determinants, including culturally embedded stigma surrounding HBV due to its associations with sexual transmission and perceived moral failing, uneven geographic distribution of preventive services that limits access for rural and lower income populations, and insufficient targeted health communication that fails to convert general awareness into consistent preventive practices [28]. The low perceived risk may reflect the relative invisibility of HBV as a community health threat following decades of pediatric vaccination success.

The finding shows that 52.5% reported fear as their dominant emotional response to a hypothetical hepatitis B infection diagnosis, and that all participants expressed concern about transmitting the infection to family members, plausibly linked to both heightened disease awareness and entrenched stigma. While fear of disease transmission can theoretically motivate care-seeking, it equally risks driving avoidance of disclosure, testing, and social contact with affected individuals’ effects well-documented in the viral hepatitis literature [29].

### 4.3. Preventive Practices

The low level of good preventive practices (16.2%) is the most notable finding of this study and represents a critical public health failure. Vaccination uptake of 26.5% and screening participation of 14.6% are markedly insufficient by any benchmark, including the WHO target of reducing new infections by 90 percent and mortality by 65 percent by 2030 among adults [30]. Alotaibi et al. reported a nearly identical screening rate of 14.8% in Saudi Arabia [8], while a study from the Riyadh region reported a higher screening rate of 35.4% alongside substantially better knowledge [14], suggesting that urban, higher-income settings may support greater preventive engagement. Internationally, the Jordanian student cohort of Jarrah et al. reported higher vaccination rates (46.6%) despite comparable knowledge levels [12], indicating that targeted structural interventions such as workplace or campus-based vaccination programs may effectively decouple practice from the knowledge–attitude complex.

Several factors may plausibly contribute to the magnitude of this knowledge–practice gap. First, the very low perceived susceptibility (14.6%) is consistent with reduced motivational impetus for preventive action, consistent with Health Belief Model constructs. Second, widespread uncertainty about the cost of HBV services (38.7% unaware) may be associated with barriers to screening engagement, even when services are nominally free. Third, low participation in health education programs (13.0%) may reflect inadequate reach of existing public health campaigns [31].

### 4.4. Sociodemographic Predictors

Participation in a hepatitis B infection educational program emerged as the single strongest modifiable predictor across all three KAP domains, with effect sizes substantially larger than those of any sociodemographic variable. This finding may suggest that the knowledge–practice gap is not a fixed attribute of this population but rather a modifiable deficit responsive to structured health education. The positive income gradient observed for knowledge and practice with participants earning 10,000–14,999 SAR demonstrating significantly higher scores across all domains is consistent with established social determinants of health frameworks, though the underlying mechanisms cannot be confirmed from the available data [32]. The absence of an independent educational attainment effect after adjustment for income and occupation suggests that these socioeconomic proxies better capture health-related behaviors in this sample than years of schooling alone.

Healthcare workers demonstrated the largest and most consistent KAP advantages across all domains, a finding consistent with occupational exposure to infection-control protocols, clinical education, and direct patient experience. Student status was independently associated with higher knowledge but not with practice, suggesting that academic exposure to health information does not automatically translate into behavioral change in the absence of additional structural support, a finding with implications for campus-based HBV prevention programs. In addition, this study reflects that polygyny was significantly negatively associated with all KAP domains, which may reflect lower health literacy, reduced engagement with formal health systems, or distinct sociocultural dynamics within polygynous households. However, due to the small subgroup size, reliance on self-report, and the potential for residual confounding by socioeconomic and regional factors, observed negative associations should be considered exploratory and require replication in appropriately designed studies [33].

The unexpected inverse association between a personal hepatitis B infection diagnosis and knowledge scores is difficult to interpret within the current cross-sectional framework; however, it may be linked to stigma-related avoidance of health information and differential socioeconomic marginalization. Therefore, dedicated longitudinal or qualitative studies are warranted to examine the behavioral, psychological, and structural factors that may contribute to this relationship [34].

### 4.5. Strengths and Limitations

This study has several strengths. The large, geographically diverse sample, the use of a validated and culturally adapted instrument, a robust multivariable analytical framework with prospectively defined modelling decisions, and adherence to STROBE reporting guidelines collectively enhance the reliability and comparability of the findings. Despite these strengths, several limitations should be acknowledged. The predominantly cross-sectional nature of the study inherently precludes causal inference, and regression findings therein should be interpreted with caution, as they reflect statistical associations rather than temporal or mechanistic relationships. Recruitment via social media using a convenience sampling approach may have introduced systematic selection bias, as suggested by the overrepresentation of females, urban residents, highly educated respondents, and individuals from the Western region residents in the final sample. These findings should therefore be interpreted with appropriate caution, recognizing that they are not nationally representative and estimated KAP levels may not accurately reflect those of more rurally located, less educated, or regionally diverse segments of the Saudi adult population.

Self-reported preventive behaviors are susceptible to social desirability bias. The notably low explanatory power of the attitude model (R^2^ = 7.2%) represents that the sociodemographic variables included in the regression framework account for only a modest fraction of the variance in attitude scores, and the identified predictors should therefore not be interpreted as definitive determinants of hepatitis B infection-related attitudes in this population. Unmeasured psychosocial constructs including health literacy, cultural norms, disease-specific stigma, and perceived social support likely account for a substantial proportion of the residual variance. Future studies incorporating validated psychosocial instruments would meaningfully strengthen the explanatory scope of this domain.

## 5. Conclusions

This study demonstrates moderate hepatitis B infection knowledge and generally positive attitudes; however, preventive practices remain substantially deficient, revealing a persistent and clinically significant knowledge–practice gap. Educational program participation was the strongest modifiable predictor across all three KAP domains, suggesting that well-designed health education interventions may improve KAP outcomes. Targeted strategies addressing the specific needs of males, lower-income groups, and polygynous households alongside systemic improvements in screening access, public awareness of service availability, and stigma reduction are essential to accelerate progress toward the WHO 2030 hepatitis B infection elimination targets and are directly aligned with Saudi Arabia’s Vision 2030 commitments to preventive medicine, population-level screening, and digital health transformation.

## Figures and Tables

**Table 1:** Sociodemographic and health-related characteristics of the study participants (n = 1278).

Characteristic	n (%) or Mean ± SD
**Age (years)**	
Mean ± SD	30.3 ± 12.4
**Sex**	
Female	778 (60.9%)
Male	500 (39.1%)
**Nationality**	
Saudi	1196 (93.6%)
Non-Saudi	82 (6.4%)
**Residence**	
Urban	1132 (88.6%)
Rural	146 (11.4%)
**Region**	
Western	812 (63.5%)
Southern	179 (14.0%)
Eastern	136 (10.6%)
Central	115 (9.0%)
Northern	36 (2.8%)
**Marital status**	
Single	701 (54.9%)
Married	529 (41.4%)
Divorced/Widowed	48 (3.8%)
**Education level**	
High school degree or lower	297 (23.2%)
Bachelor’s/Diploma degree	893 (69.9%)
Postgraduate studies	88 (6.9%)
**Monthly household income (SAR)**	
<5000 (<$1330)	328 (25.7%)
5000–9999 ($1330–$2660)	248 (19.4%)
10,000–14,999 ($2660–$4000)	337 (26.4%)
≥15,000 (≥$4000)	365 (28.6%)
**Work sector**	
Healthcare sector	167 (13.1%)
Student	481 (37.6%)
Other employed *	474 (37.1%)
Unemployed	156 (12.2%)
**Smoking status**	
Never smoker	1070 (83.7%)
Current smoker	112 (8.8%)
Ex-smoker	96 (7.5%)

Abbreviations: SAR, Saudi Arabian Riyal; SD, standard deviation. * ‘Other employed’ comprises educational sector (n = 304, 23.8%), military sector (n = 84, 6.6%), administrative sector (n = 40, 3.1%), industrial sector (n = 23, 1.8%), handicrafts/freelance (n = 18, 1.4%), and agricultural sector (n = 5, 0.4%).

**Table 2:** Hepatitis B diagnosis, family history, and prior educational program participation (n = 1278).

Characteristic	n (%)
**Hepatitis B infection diagnosis**	
No	1248 (97.7%)
Yes	30 (2.3%)
**Family history of hepatitis B infection**	
No	1170 (91.5%)
Yes	108 (8.5%)
**Prior participation in HBV educational program**	
No	1173 (91.8%)
Yes	105 (8.2%)

*Abbreviations: HBV, hepatitis B virus.*

**Table 3:** Knowledge of hepatitis B infection among study participants (n = 1278).

Knowledge Item (Correct Answer)	Correct n (%)	Incorrect n (%)
**General awareness**		
Ever heard of hepatitis (Yes)	979 (76.6%)	299 (23.4%)
Ever heard of hepatitis B specifically (Yes)	833 (65.2%)	445 (34.8%)
**Disease characteristics**		
Hepatitis B infection is a viral disease (Yes)	620 (48.5%)	658 (51.5%)
Hepatitis B infection affects liver function (Yes)	833 (65.2%)	445 (34.8%)
Hepatitis B infection can cause liver cancer (Yes)	483 (37.8%)	795 (62.2%)
Hepatitis B infection can affect any age group (Yes)	656 (51.3%)	622 (48.7%)
**Symptoms**		
Early symptoms resemble cold/flu (Yes)	1021 (79.9%)	257 (20.1%)
Jaundice is a common symptom (Yes)	613 (48.0%)	665 (52.0%)
Nausea, vomiting, and loss of appetite are common (Yes)	488 (38.2%)	790 (61.8%)
Some patients may remain asymptomatic (Yes)	454 (35.5%)	824 (64.5%)
**Transmission routes**		
Via unsterilized needles or surgical instruments (Yes)	632 (49.5%)	646 (50.5%)
Via contaminated blood or blood products (Yes)	662 (51.8%)	616 (48.2%)
Via barber blades or piercings (Yes)	572 (44.8%)	706 (55.2%)
Via unsafe sexual contact (Yes)	491 (38.4%)	787 (61.6%)
Via mother-to-child transmission (Yes)	421 (32.9%)	857 (67.1%)
NOT via contaminated food or water (No)	906 (70.9%)	372 (29.1%)
**Prevention and treatment**		
Hepatitis B infection is treatable (Yes)	759 (59.4%)	519 (40.6%)
Cannot be self-cured by the body (No)	1090 (85.3%)	188 (14.7%)
Vaccination is available (Yes)	704 (55.1%)	574 (44.9%)
No specific diet required for treatment (No)	905 (70.8%)	373 (29.2%)
**Overall knowledge score**		
Mean ± SD (out of 20 items)	11.1 ± 3.7	
Good knowledge * (score ≥ 11, ≥55%)	693 (54.2%)	
Poor knowledge (score < 11)	585 (45.8%)	

* Knowledge was scored as the sum of correct binary responses across 20 items (range: 0–20). Good knowledge was defined as a score ≥ 11 (≥55%), consistent with the adapted Haq et al. [9] instrument.

**Table 4:** Attitudes toward hepatitis B infection among study participants (n = 1278).

Attitude Item	n (%)	Scoring *
**Perceived susceptibility**		
Believe they could contract hepatitis B infection (Yes)	186 (14.6%)	Positive
Does not perceive personal risk (No)	1092 (85.4%)	Negative
**Emotional response if diagnosed**		
Fear (health-motivated response)	671 (52.5%)	Positive
Sadness	342 (26.8%)	Neutral
Surprise	232 (18.2%)	Neutral
Shame	33 (2.6%)	Negative
**Disclosure and healthcare-seeking behavior**		
Would inform a responsible person (physician/family)	1185 (92.7%)	Positive
Would seek health facility if symptomatic	1161 (90.8%)	Positive
Would seek care promptly (soon after recognizing symptoms)	980 (76.7%)	Positive
**Perceived cost of care**		
Perceives service as free or reasonably priced	784 (61.3%)	Positive
Unaware of cost	494 (38.7%)	Negative
**Primary concern if diagnosed**		
Fear of transmitting infection to family members	1278 (100.0%)	—
**Overall attitude score**		
Mean ± SD (out of 7 items)	4.9 ± 1.1	
Positive attitude (score ≥ 5, ≥71.4%)	876 (68.5%)	
Negative attitude (score < 5)	402 (31.5%)	

* Attitude items were scored using a binary framework (positive = 1, negative/neutral = 0) adapted from Haq et al. [9]; a total score ≥ 5 (≥71.4%) defined a positive attitude.

**Table 5:** Preventive practices related to hepatitis B infection among study participants (n = 1278).

Practice Item * (Correct Answer)	Good Practice n (%)	Poor Practice n (%)
**Preventive health services**		
Screened for hepatitis B infection (Yes)	186 (14.6%)	1092 (85.4%)
Vaccinated against hepatitis B infection (Yes)	338 (26.5%)	940 (73.5%)
**Situational preventive behaviors**		
Would request a new syringe before use (Yes)	840 (65.7%)	438 (34.3%)
Would request blood screening before transfusion (Yes)	733 (57.4%)	545 (42.6%)
Would request safe barbering or piercing practices (Yes)	862 (67.4%)	416 (32.6%)
**Response to diagnosis**		
Would seek further investigation/treatment if diagnosed (Yes)	1000 (78.2%)	278 (21.8%)
Would not avoid contact with hepatitis B infection patients (No)	733 (57.4%)	545 (42.6%)
**Health education engagement**		
Ever participated in any health education program (Yes)	166 (13.0%)	1112 (87.0%)
**Overall practice score**		
Mean ± SD (out of 8 items)	3.8 ± 1.8	
Good practice (score ≥ 6, ≥75%)	207 (16.2%)	
Poor practice (score < 6)	1071 (83.8%)	

* Practice was scored as the sum of correct binary responses across 8 items (range: 0–8). Good practice was defined as a score ≥ 6 (≥75%), consistent with the adapted Haq et al. [9] instrument.

**Table 6:** Multivariable linear regression: predictors of hepatitis B infection knowledge, attitude, and practice scores (n = 1278).

Predictor	Knowledge Score			Attitude Score			Practice Score		
	**β**	**95% CI**	***p***	**β**	**95% CI**	***p***	**β**	**95% CI**	***p***
Age	**+0.06**	**0.03–0.08**	**<0.001**	**+0.01**	**0.001–0.016**	**0.021**	**+0.02**	**0.008–0.031**	**<0.001**
**Sex (reference: Female)**									
Male	**−1.06**	**−1.49–−0.64**	**<0.001**	−0.12	−0.25–0.02	0.098	**−0.34**	**−0.54–−0.13**	**0.002**
**Nationality (reference: Non-Saudi)**									
Saudi	+0.57	−0.21–+1.35	0.153	+0.05	−0.20–+0.30	0.700	−0.12	−0.50–+0.26	0.526
**Residence (reference: Rural)**									
Urban	−0.03	−0.61–+0.56	0.933	−0.11	−0.30–+0.08	0.255	−0.003	−0.29–+0.28	0.982
**Marital status (reference: Single)**									
Married	**+0.75**	**0.13–1.36**	**0.017**	**+0.28**	**0.08–0.48**	**0.006**	+0.28	−0.02–+0.58	0.067
Divorced/Widowed	+0.25	−0.88–+1.38	0.663	+0.10	−0.26–+0.46	0.585	+0.15	−0.40–+0.70	0.589
**Polygyny (reference: No)**									
Yes	**−0.96**	**−1.78–−0.13**	**0.023**	**−0.50**	**−0.76–−0.23**	**<0.001**	**−0.54**	**−0.95–−0.14**	**0.009**
**Education level (reference: High school or lower)**									
Bachelor’s/Diploma	+0.30	−0.16–+0.77	0.202	−0.04	−0.19–+0.11	0.603	+0.06	−0.17–+0.29	0.584
Postgraduate	+0.82	−0.06–+1.69	0.067	−0.06	−0.35–+0.22	0.658	+0.08	−0.35–+0.51	0.716
**Monthly income, SAR (reference: <5000)**									
5000–9999	+0.53	−0.05–+1.10	0.071	+0.002	−0.18–+0.19	0.980	+0.28	−0.003–+0.56	0.053
10,000–14,999	**+0.81**	**0.25–1.38**	**0.005**	**+0.23**	**0.05–0.41**	**0.013**	**+0.56**	**0.28–0.83**	**<0.001**
≥15,000	**+0.86**	**0.31–1.41**	**0.002**	+0.10	−0.08–+0.28	0.268	**+0.39**	**0.13–0.66**	**0.004**
**Work sector (reference: Unemployed)**									
Healthcare	**+2.54**	**1.75–3.33**	**<0.001**	**+0.27**	**0.02–0.52**	**0.038**	**+0.90**	**0.52–1.29**	**<0.001**
Student	**+1.16**	**0.44–1.88**	**0.002**	+0.16	−0.07–+0.39	0.175	−0.01	−0.36–+0.34	0.944
Other employed	−0.03	−0.69–+0.62	0.919	−0.01	−0.22–+0.20	0.946	−0.20	−0.52–+0.12	0.215
**Smoking status (reference: Never smoker)**									
Ex-smoker	−0.16	−0.91–+0.59	0.678	−0.07	−0.31–+0.17	0.573	+0.13	−0.24–+0.50	0.489
Current smoker	+0.03	−0.67–+0.73	0.929	−0.05	−0.28–+0.17	0.657	+0.003	−0.34–+0.35	0.984
**Educational program participation (reference: No)**									
Yes	**+1.89**	**1.20–2.58**	**<0.001**	**+0.47**	**0.25–0.69**	**<0.001**	**+1.43**	**1.09–1.77**	**<0.001**
**Hepatitis B infection diagnosis (reference: No)**									
Yes	**−1.41**	**−2.72–−0.11**	**0.034**	−0.41	−0.83–+0.006	0.053	+0.10	−0.53–+0.74	0.753
**Family history of hepatitis B infection (reference: No)**									
Yes	**+1.69**	**0.98–2.39**	**<0.001**	+0.10	−0.13–+0.33	0.395	**+0.87**	**0.52–1.21**	**<0.001**
**R^2^ (Adj. R^2^)**	**17.4% (16.1%)**			**7.2% (5.7%)**			**18.5% (17.2%)**		

## Data Availability

The datasets generated and analyzed during the current study are available from the corresponding author upon reasonable request.

## Abbreviations

The following abbreviations are used in this manuscript:

HBV	Hepatitis B virus
KAP	Knowledge, Attitudes, and Practices
WHO	World Health Organization
HCC	Hepatocellular Carcinoma
CI	Confidence Interval
β	Beta Coefficient
SD	Standard Deviation
IQR	Interquartile Range
R^2^	Coefficient of Determination
Adj. R^2^	Adjusted Coefficient of Determination
SAR	Saudi Arabian Riyal
REC	Research Ethics Committee
AI	Artificial Intelligence
STROBE	Strengthening the Reporting of Observational Studies in Epidemiology

## References

[1] World Health Organization (2024). Global Hepatitis Report 2024: Action for Access in Low- and Middle-Income Countries.

[2] World Health Organization (2022). Global Health Sector Strategies on, Respectively, HIV, Viral Hepatitis and Sexually Transmitted Infections for the Period 2022–2030.

[3] Al-Faleh F.Z., Al-Jeffri M., Ramia S., Al-Rashed R., Arif M., Rezeig M., AI-Toraif I., Bakhsh M., Mishkkhas A., Makki O. (1999). Seroepidemiology of hepatitis B virus infection in Saudi children 8 years after a mass hepatitis B infection vaccination programme. J. Infect..

[4] Memish Z.A., Knawy B.A., El-Saed A. (2010). Incidence trends of viral hepatitis over eight years of surveillance in Saudi Arabia. Int. J. Infect. Dis..

[5] Alghamdi I.G., Alghamdi R.M., Alghamdi M.S., Alghamdi A.M., Alghamdi M.I., Alghamdi Z.I., Alghamdi K.S. (2023). Epidemiology of Hepatitis B in Saudi Arabia from 2006 to 2021. Hepat. Med..

[6] Abdo A.A., Sanai F.M., Al-Faleh F.Z. (2012). Epidemiology of viral hepatitis in Saudi Arabia: Are we off the hook?. Saudi J. Gastroenterol..

[7] Wang Q., Wu Y., Wang D., Lai X., Tan L., Zhou Q., Duan L., Lin R., Wang X., Zheng F. (2023). The impacts of knowledge and attitude on behavior of antibiotic use for the common cold among the public and identifying the critical behavioral stage: Based on an expanding KAP model. BMC Public Health.

[8] Balegha A.N., Yidana A., Abiiro G.A. (2021). Knowledge, attitude and practice of hepatitis B infection prevention among nursing students in the Upper West Region of Ghana: A cross-sectional study. PLoS ONE.

[9] Garg M., Sridhar B., Katyal V., Goyal S. (2023). Assessment of Knowledge, Attitude, and Practices (KAP) Toward Hepatitis B Infection, Its Prevention, and Vaccination Among Health Care Workers. Cureus.

[10] Jeng W.J., Papatheodoridis G.V., Lok A.S.F. (2023). Hepatitis B infection. Lancet.

[11] Almalki F., Alraffah Y.M., Alasiri R.A., Dhafar M.W., Albogami F.M., Alhazmi M.N., Alyazidi A.M., Alharbi L.A., Alotaibi M.E. (2025). Knowledge, Attitude and Practice Towards Hepatitis B Infection and HBV Vaccine Among the Healthy Population in Makkah, Saudi Arabia. Infect. Drug Resist..

[12] Al-Thaqafy M.S., Balkhy H.H., Memish Z., Makhdom Y.M., Ibrahim A., Al-Amri A., Al-Thaqafi A. (2012). Improvement of the low knowledge, attitude and practice of hepatitis B virus infection among Saudi national guard personnel after educational intervention. BMC Res. Notes.

[13] Alotaibi B.S., Althobaiti M.A., Hazazi A.Y., Hazazi S.Y., Nassir R.A., Alhaddad M.S., Abdelwahab S.F. (2021). Exploration of Knowledge, Attitude, and Practice Among Residents of Saudi Arabia Toward Hepatitis Viruses. Inquiry.

[14] Abukaram T.M., Alwan M., Alanazi A.K., Habra S.M., Almalik A.M., Alanazi S.S., Alali N.M., Alnowaisser L., Alotaibi R., Farooqi W. (2025). Awareness of Hepatitis B Among the General Population in Riyadh, Saudi Arabia. Cureus.

[15] Chen F., Li Q., Xu X., Wang F. (2023). Clinical characteristics and risk factors of hepatitis B virus-related cirrhosis/hepatocellular carcinoma: A single-center retrospective study. Liver Res..

[16] Ryani M.A. (2025). Hepatitis Management in Saudi Arabia: Trends, Prevention, and Key Interventions (2016–2025). Medicina.

[17] Al-Hazmi A.H. (2014). Knowledge, attitudes and practice of primary health care physicians towards hepatitis B virus in Al-Jouf province, Saudi Arabia. BMC Res. Notes.

[18] Homoud A.H. (2014). Knowledge, attitudes and practice of primary healthcare physicians concerning the occupational risks of hepatitis B virus in Al Jouf Province, Saudi Arabia. J. Infect. Public Health.

[19] Chowdhury S., Mok D., Leenen L. (2021). Transformation of health care and the new model of care in Saudi Arabia: Kingdom’s Vision 2030. J. Med. Life.

[20] Alasiri A.A., Mohammed V. (2022). Healthcare Transformation in Saudi Arabia: An Overview Since the Launch of Vision 2030. Health Serv. Insights.

[21] Alghamdi M., Alghamdi A.S., Aljedai A., Khathlan A.A., Masri N.A., Qutub A., Quaiz M.A., Sanai F., Subahi G., Sulimani S. (2021). Revealing Hepatitis B Virus as a Silent Killer: A Call-to-Action for Saudi Arabia. Cureus.

[22] Alarifi A.M., Alshahrani N.Z., Jokhdar H., Asiri A.M. (2025). Advancing Health Through Sustainable Development Goals-Saudi Arabia’s Mid-Journey Progress and Insights. J. Epidemiol. Glob. Health.

[23] Haq N.U., Hassali M.A., Shafie A.A., Saleem F., Farooqui M., Aljadhey H. (2012). A cross-sectional assessment of knowledge, attitude and practice towards Hepatitis B among healthy population of Quetta, Pakistan. BMC Public Health.

[24] Al-Hanawi M.K., Angawi K., Alshareef N., Qattan A.M.N., Helmy H.Z., Abudawood Y., Alqurashi M., Kattan W.M., Kadasah N.A., Chirwa G.C. (2020). Knowledge, Attitude and Practice Toward COVID-19 Among the Public in the Kingdom of Saudi Arabia: A Cross-Sectional Study. Front. Public Health.

[25] Al-Hanawi M.K., Alsharqi O., Almazrou S., Vaidya K. (2018). Healthcare finance in the Kingdom of Saudi Arabia: A qualitative study of householders’ attitudes. Appl. Health Econ. Health Policy.

[26] Alaridah N., Joudeh R.M., Al-Abdallat H., Jarrar R.F., Ismail L., Jum’ah M., Alnajjar Z., Alzyoud E., Battah Z., Battah A. (2023). Knowledge, attitude, and practices toward hepatitis B infection among healthcare students: A nationwide cross-sectional study in Jordan. Int. J. Environ. Res. Public Health.

[27] Rosenstock I.M. (1974). The Health Belief Model and preventive health behavior. Health Educ. Monogr..

[28] Mokaya J., McNaughton A.L., Burbridge L., Maponga T., O’Hara G., Andersson M., Seeley J., Matthews P.C. (2018). A blind spot? Confronting the stigma of hepatitis B virus (HBV) infection—A systematic review. Wellcome Open Res..

[29] Mak W.W.S., Mo P.K.H., Cheung R.Y.M., Woo J., Cheung F.M., Lee D. (2006). Comparative stigma of HIV/AIDS, SARS, and tuberculosis in Hong Kong. Soc. Sci. Med..

[30] World Health Organization (2016). Global Health Sector Strategy on Viral Hepatitis 2016–2021. Towards Ending Viral Hepatitis (No. WHO/HIV/2016.06).

[31] Mukhtar N.A., Evon D.M., Yim C., Lok A.S., Lisha N., Lisker-Melman M., Hassan M., Janssen H.L.A., Khalili M. (2021). Patient Knowledge, Beliefs and Barriers to Hepatitis B Care: Results of a Multicenter, Multiethnic Patient Survey. Dig. Dis. Sci..

[32] Braveman P., Gottlieb L. (2014). The social determinants of health: It’s time to consider the causes of the causes. Public Health Rep..

[33] Shaiful Bahari I., Norhayati M.N., Nik Hazlina N.H., Mohamad Shahirul Aiman C.A.A., Nik Muhammad Arif N.A. (2021). Psychological impact of polygamous marriage on women and children: A systematic review and meta-analysis. BMC Pregnancy Childbirth.

[34] Freeland C., Mendola L., Cheng V., Cohen C., Wallace J. (2022). The unvirtuous cycle of discrimination affecting people with hepatitis B: A multi-country qualitative assessment of key-informant perspectives. Int. J. Equity Health.

